# Effect of methanol extract of Salviae miltiorrhizae Radix in high-fat diet-induced hyperlipidemic mice

**DOI:** 10.1186/s13020-017-0150-0

**Published:** 2017-10-13

**Authors:** Chiyeon Lim, Sehyun Lim, Byoungho Lee, Buyeo Kim, Suin Cho

**Affiliations:** 1 College of Medicine, Dongguk University, Ilsandong-gu, Gyeonggi-Do 10326 Republic of Korea; 2 School of Public Health, Far East University, Chungbuk, 27601 Republic of Korea; 3 Kyunghee Naseul Korean Medicine Clinic, Bucheon-si, Gyeonggi-do 14548 Republic of Korea; 4 Department of Medical Research, Korea Institute of Oriental Medicine, Daejeon, 34054 Republic of Korea; 5 School of Korean Medicine, Yangsan Campus of Pusan National University, Yangsan-si, 50612 Republic of Korea

**Keywords:** Salviae miltiorrhizae Radix, Hyperlipidemia, Cardiovascular diseases

## Abstract

**Background:**

The dried root of *Salvia miltiorrhiza*, Salviae miltiorrhizae Radix (SR), is one of the most popular medicinal herbs in Asian countries such as China and Korea. In Asian traditional medicine, SR is considered to have a bitter flavor, be slightly cold in nature, and exert therapeutic actions in the heart and liver meridians. Thus, SR has been used to control symptoms related to cardiovascular diseases. Hyperlipidemia is recognized as the main cause of cerebrovascular and heart diseases; consequently, therapeutic strategies for hyperlipidemia have been widely studied. In this study, the effects and molecular targets of methanol extract of SR (SRme) in hyperlipidemic mice were investigated.

**Methods:**

High-fat diet was fed to mice to induce hyperlipidemia, and measurement of blood cholesterol and triglycerides were conducted to evaluate the effect of SRme on hyperlipidemic mice, and gene expression in mice liver was analyzed to identify key molecules which could be potential targets for developing anti-hyperlipidemic herbal medicines.

**Results:**

There was no significant effect on the body weight gain of hyperlipidemic mice, but the triglyceride content in blood was significantly reduced by the administration of SRme to hyperlipidemic mice. Proteins such as minichromosome maintenance (Mcm) family which play a key role in DNA replication were identified as molecular targets in the amelioration of hyperlipidemia.

**Conclusions:**

SRme ameliorated hyperlipidemia in high-fat diet fed mice by inhibiting increase of blood serum level of triglycerides. And several proteins such as Mcm proteins were deduced to be molecular targets in treating hyperlipidemia.

**Electronic supplementary material:**

The online version of this article (doi:10.1186/s13020-017-0150-0) contains supplementary material, which is available to authorized users.

## Background

Urbanized living environments and excessive nutritional intake have resulted in the recent increase of various metabolic diseases such as diabetes, hypertension, hyperlipidemia, and cardiovascular diseases [1, 2]. After cancer, cerebrovascular disease and heart disease are the second and third most common causes of death in Korea [3–5].

Hyperlipidemia is recognized as a direct cause of cerebrovascular disease and heart disease; thus, diverse therapeutic strategies for hyperlipidemia have been studied [6]. A direct correlation between diabetes and hyperlipidemia as risk factors has been reported [1, 7, 8]. Indeed, cardiovascular disease is the leading cause of death in diabetic patients, and it is known that 31–34% of diabetic patients also have coronary artery disease [1, 2, 8].

Salviae miltiorrhizae Radix (SR), the dried root of *Salvia miltiorrhiza*, is one of the most popularly used medicinal herbs. Recently, it has received increasing attention for the treatment and prevention of cardiovascular system disorders [9–12]. The major bioactive constituents of SR can be classified into hydrophilic components, such as salvianolic acids, and lipophilic components, such as diterpenoid tanshinones [12, 13].

As herbal extracts such as SR contain many kinds of bioactive compounds, and the selection of extraction methods of herbal preparations may affect results of pharmaceutical research of herbal medicines. Thus its extraction process is one of the most important steps in research of herbal resources. Recently, pharmaceutical network studies are conducted to identify the molecular targets which play key role on the effects of herbal medicines. But there are still many unclear data from pharmaceutical network studies due to the diversity of extraction methods on pharmaceutical researches which were used to support the pharmaceutical network studies.

Salviae miltiorrhizae Radix has been reported to affect coronary heart disease [14], ischemia/reperfusion-induced myocardial injury [15], cancer [16], metabolic syndrome [10], Alzheimer’s disease [17], and osteoporosis [12]. Several research articles have reported the effects of SR on diet-induced hyperlipidemia in rats; in one study, rats were administered SR extract for 4 weeks, which resulted in a significant decrease in serum lipid levels [18–20]. Recently, we reported the anti-inflammatory and anti-hyperlipidemic effects of SR, which were thought to be mediated through the anti-oxidative effects of the extract [19, 21]. In the above study, we modified a mouse model for hyperlipidemia experimentation, orally administered the herbal extracts mixed with chow for rodents, and determined the appropriate dosages for mice.

As SR exerts various pharmacological activities, it has great potential as a pharmacological agent [12, 15, 18]. In this study, we aimed to confirm the anti-hyperlipidemic effects in mice and to determine the molecular targets of SR.

In order to investigate the effects and the molecular targets of the methanol extract of SR (SRme) in high-fat diet induced hyperlipidemic mice, we monitored changes in body weight and the blood serum contents of total cholesterol, high-density lipoprotein (HDL)-cholesterol, and triglycerides. The extent of accumulation of lipid peroxide owing to lipid metabolism disorder was also evaluated through measurement of malondialdehyde (MDA) level. In addition, after the evaluation of gene expression in hepatic tissues, the target proteins of SRme were identified by using a protein interaction database.

## Methods

### Animals

Six-week-old male ICR mice (SAMTAKO, Korea), weight 20–25 g, were used for the experiments involving the induction of hyperlipidemia. The mice were adapted to the laboratory environment (room temperature: 24 ± 2 °C; humidity: 55 ± 5%; 12-h light/dark cycle) for a minimum of 1 week with a sufficient supply of solid feed and water. The experimental protocol involving animals was approved by the ethics committee of PNU (Pusan National University; Approval Number PNU-2013-0311). The Minimum Standards of Reporting Checklist (Additional file 1) contains details of the experimental design, statistics, and resources used in this study.

### Preparation of SRme

The SR used in this study was purchased from an authorized pharmaceutical company (Gwangmyoung Co., Korea) and authenticated by Dr. Cho (School of Korean Medicine, Pusan National University, Yangsan, Korea). Fingerprinting data of the SR was kindly provided from Gwangmyoung Co., and the data are shown as Additional file 2: Figure S1. A voucher specimen (No. SM14-0611) was deposited in the low temperature room (4 °C) of the laboratory. SR (500 g) was immersed in methanol at room temperature for 5 days; this process was repeated twice and a total of 58.4 g of dry extract was obtained (11.7% yield).

### Induction of hyperlipidemia and classification of experimental groups

**Fig. 1:**
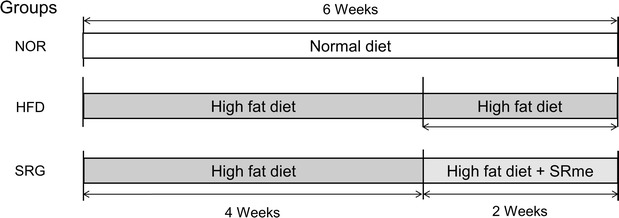
Design of hyperlipidemia induction. The mice were fed a normal diet or high-fat diet, based on their allocated group, for 6 weeks. SRme mixed with high-fat diet was fed to the mice in the SRme administration group (SRG) for the final 2 weeks. NOR: naive mice (n = 8), HFD: hyperlipidemic mice (n = 8), SRG: SRme-treated hyperlipidemic mice (n = 8)

To induce hyperlipidemia, we fed a high-fat diet to the mice in the control group (HFD) and the SRme-treated group (SRG) for 4 weeks. Mice in the normal group (NOR) were supplied general feed. On the fifth week of the experiment, high-fat diet-fed mice were randomly allocated to HFD and SRG based on body weight. From the fifth week of the experiment, SRG mice, which received a high-fat diet with SRme, and HFD mice, which received a high-fat diet only, were fed for an additional 2 weeks. The rodent chow was custom made by Daol Biotech (Daejeon, Korea). The composition of main ingredients and nutrition facts are given in Tables 1, 2. The schematic design of this study is shown in Fig. 1.

**Table 1:** Experimental groups and compositions of normal and high fat diet

Main ingredients	Diet (g/kg)		
	Normal	High fat	High fat + SRme
Casein	200	200	200
Sucrose	172.8	172.8	172.8
Dextrose	100	100	100
Soybeal oil	–	25	25
Lard^a^	–	177.5	177.5
Cholesterol	–	12.9	12.9
Cholic acid	–	4.3	4.3
SRme	–	–	1

^a^Typical analysis of cholesterol in lard = 0.95 mg/g

**Table 2:** Gram percentage of main nutrition facts

Compounds	Diet (g%)		
	Normal	High fat	High fat + SRme
Protein	28	28	28
Carbohydrate	25	25	25
Fat	5	24	24
Crude fiber	4	4	4
Mineral mix	5	5	5
Vitamin mix	2	2	2
Water	12	12	12
Total kcal/g	3.5	4.7	4.7

### Harvesting liver tissues, preparation for gene expression analysis, and MDA measurement

After the experimental animals were sacrificed, the liver tissue was excised and blood was removed using cold (4 °C) perfusion solution (130 mM NaCl, 5 mM KCl, and 10 mM Tris–HCl, pH 7.4). In order to observe gene expression, total RNA was isolated by using a Qiagen RNeasy Kit (Qiagen Korea Ltd) in accordance with the manufacturer’s instructions. An Agilent microarray containing approximately 45,000 oligo-spots (Agilent Technologies Co.) was used for hybridization. In comparison with RNA from NOR mice as a reference, we considered genes that showed a greater than threefold upregulation or downregulation. Gene expression folds based on NOR were shown as Additional file 3. Hierarchical clusters of genes were analyzed using a multiple experiment viewer (MeV ver. 4.9, mev.tm4.org) and a functional protein association networks database (STRING, https://string-db.org) was applied for interaction network analysis.

To measure the MDA levels, a Stadie-Riggs microtome (Tomas Co. USA) was used to prepare tissue slices approximately 1-mm wide and 0.3–0.5-mm thick, with a horizontal length and a vertical length of 1 cm each. Phosphoric acid (3 ml) and 0.6% thiobarbituric acid solution were added to the slices and boiled for 60 min. Finally, 1-butanol (4 ml) was added, thoroughly mixed, and centrifuged at 800×*g* for 25 min. The absorbance of the supernatant of the mixed solution was measured at 534 and 510 nm.

### Blood collection and measurement of blood cholesterol and triglycerides

At the end of the 2-week drug administration period, blood was collected from the mouse abdominal vein. After the collected blood was centrifuged at 5000×*g* for 20 min, the supernatant was removed for the measurements of blood cholesterol and triglyceride levels. Serum total cholesterol, HDL-cholesterol, and triglycerides were measured by using measurement kits (FUJIFILM, Japan).

### Statistical analysis

To perform the statistical analyses on the experimental material, SigmaPlot ver. 12 (SigmaStat, USA) was used. The experimental results were expressed as the mean ± standard deviation (mean ± SD) and statistical significance between groups was determined by using one-way ANOVA followed by Tukey’s post hoc analysis. Values of P < 0.05 were considered to be statistically significant.

## Results

### Effect on body weight

**Fig. 2:**
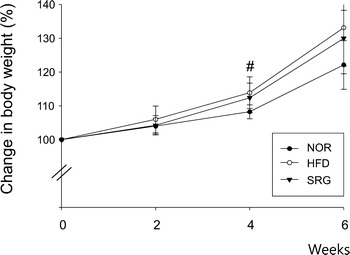
Effects of SRme on body weight in hyperlipidemic mice. Body weight was measured every 2 weeks. NOR: naive mice (n = 8), HFD: hyperlipidemic mice (n = 8), SRG: SRme-treated hyperlipidemic mice (n = 8). The values are presented as the mean ± SD

A slight increase in body weight was observed in HFD mice in comparison to that of NOR mice over the 4-week hyperlipidemia induction period, but in the additional 2-week period, there was no statistically significant difference among the groups (Fig. 2). There was also no difference between the groups in food intake during the experimental periods (data not shown).

### Effect on serum lipid content

**Fig. 3:**
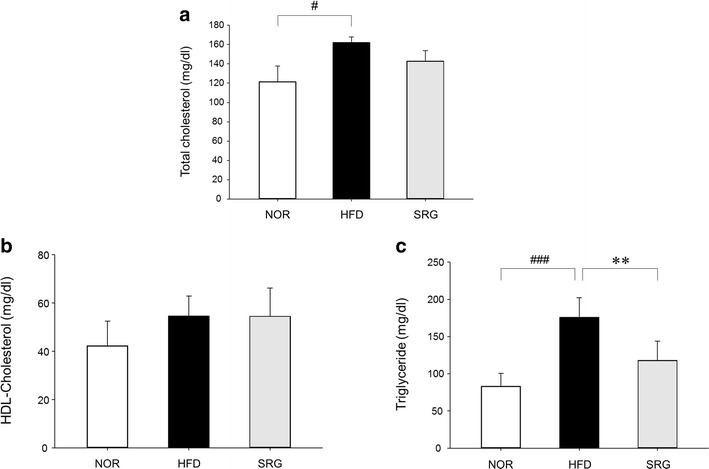
Effects of SRme on the levels of total cholesterol, HDL-cholesterol, and triglycerides in hyperlipidemic mice. The levels of total cholesterol (**a**), HDL-cholesterol (**b**), and triglycerides (**c**) in serum were measured spectrophotometrically. NOR: naive mice (n = 8), HFD: hyperlipidemic mice (n = 8), SRG: SRme-treated hyperlipidemic mice (n = 8). The values are presented as the mean ± SD. ^#^ P < 0.05, ^###^ P < 0.001 vs NOR; *P < 0.05 in comparison with CON

The total cholesterol content in blood was significantly different between NOR and HFD mice (121.38 ± 16.42 and 162.00 ± 6.09 mg/dl, respectively). However, the total cholesterol content in SRG mice was 142.88 ± 10.80 mg/dl, which was not significantly different from that of HFD mice (Fig. 3a).

The HDL-cholesterol content in mouse blood was not observed to be significantly different in any groups (Fig. 3b).

A statistically significant increase was observed when NOR and HFD mice were compared (83.00 ± 17.56 and 175.88 ± 26.07 mg/dl, respectively). In SRG mice, the value was 117.75 ± 26.26, which was also significantly different from that in HFD mice (Fig. 3c).

### Changes in lipid peroxide content in liver tissue

**Fig. 4:**
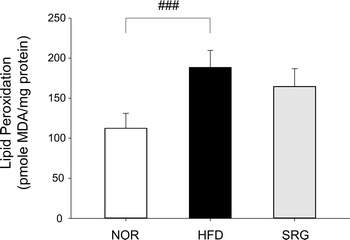
Effects of SRme on lipid peroxidation levels in hyperlipidemic mice. Lipid peroxidation in liver tissues was measured spectrophotometrically. NOR: naive mice (n = 8), HFD: hyperlipidemic mice (n = 8), SRG: SRme-treated hyperlipidemic mice (n = 8). The values are presented as the mean ± SD

Level of MDA, a lipid peroxide, in mouse liver tissue, showed a significant increase in hyperlipidemic HFD mice in comparison to that in the non-hyperlipidemic NOR mice (188.5 ± 21.3 pmol MDA/mg protein and 112.6 ± 18.3 pmol MDA/mg protein, respectively). However, SRG mice showed no significant change compared with HFD mice (164.6 ± 22.2 pmol MDA/mg protein) (Fig. 4).

### Expression profile of genes

**Fig. 5:**
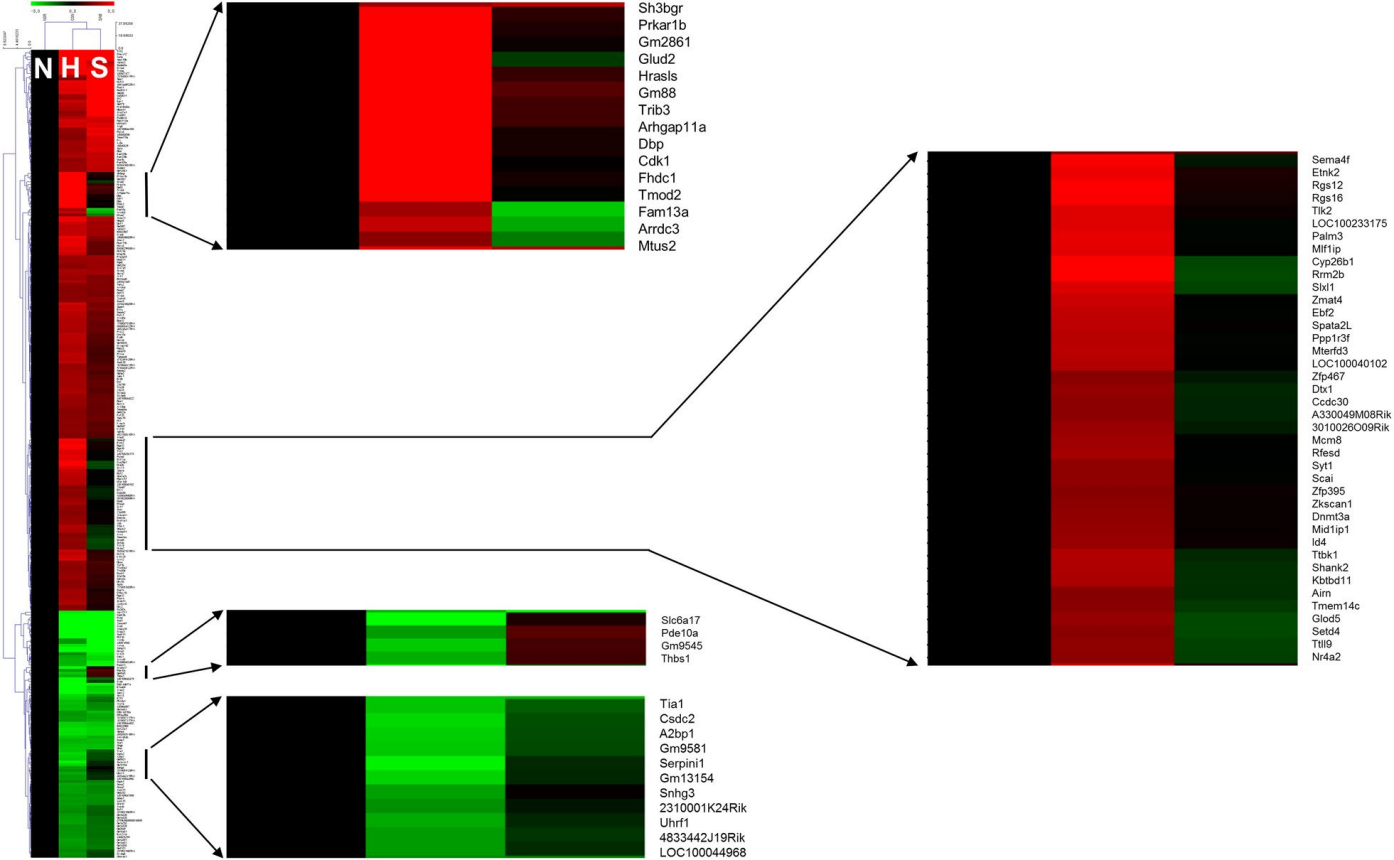
Effects of SRme on gene expression patterns in liver tissue of hyperlipidemic mice. To identify the genes using the quantitative analysis and expression clustering, MeV ver. 4.0 software was used. Genes colored red were upregulated compared with NOR mice (N); genes colored green were downregulated compared with NOR mice. N: naive mice, H: hyperlipidemic mice, S: SRme-treated hyperlipidemic mice

**Fig. 6:**
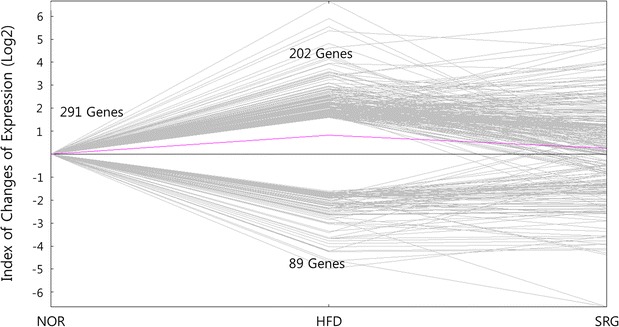
Line plot of alteration of gene expression liver tissues in hyperlipidemic mice. The resultant SRme-responsive genes are plotted as log values for each differentially expressed gene. NOR: naive mice, HFD: hyperlipidemic mice, SRG: SRme-treated hyperlipidemic mice

The analysis of the expression pattern of genes in mice liver revealed that a total of 291 genes showed at least threefold change in HFD mice as compared to the values in NOR mice. These changed genes were hierarchically clustered (Additional file 4: Figure S2), as shown in Fig. 5. It is clear that the expression of 291 genes was significantly changed in the livers of hyperlipidemic HFD mice in comparison to that in NOR mice. From the altered genes, we selected 71 genes whose expression was restored by SRme administration, based on hierarchical clustering using MeV software (Fig. 5). The trends in alteration and restoration of the genes are shown in Fig. 6.

**Fig. 7:**
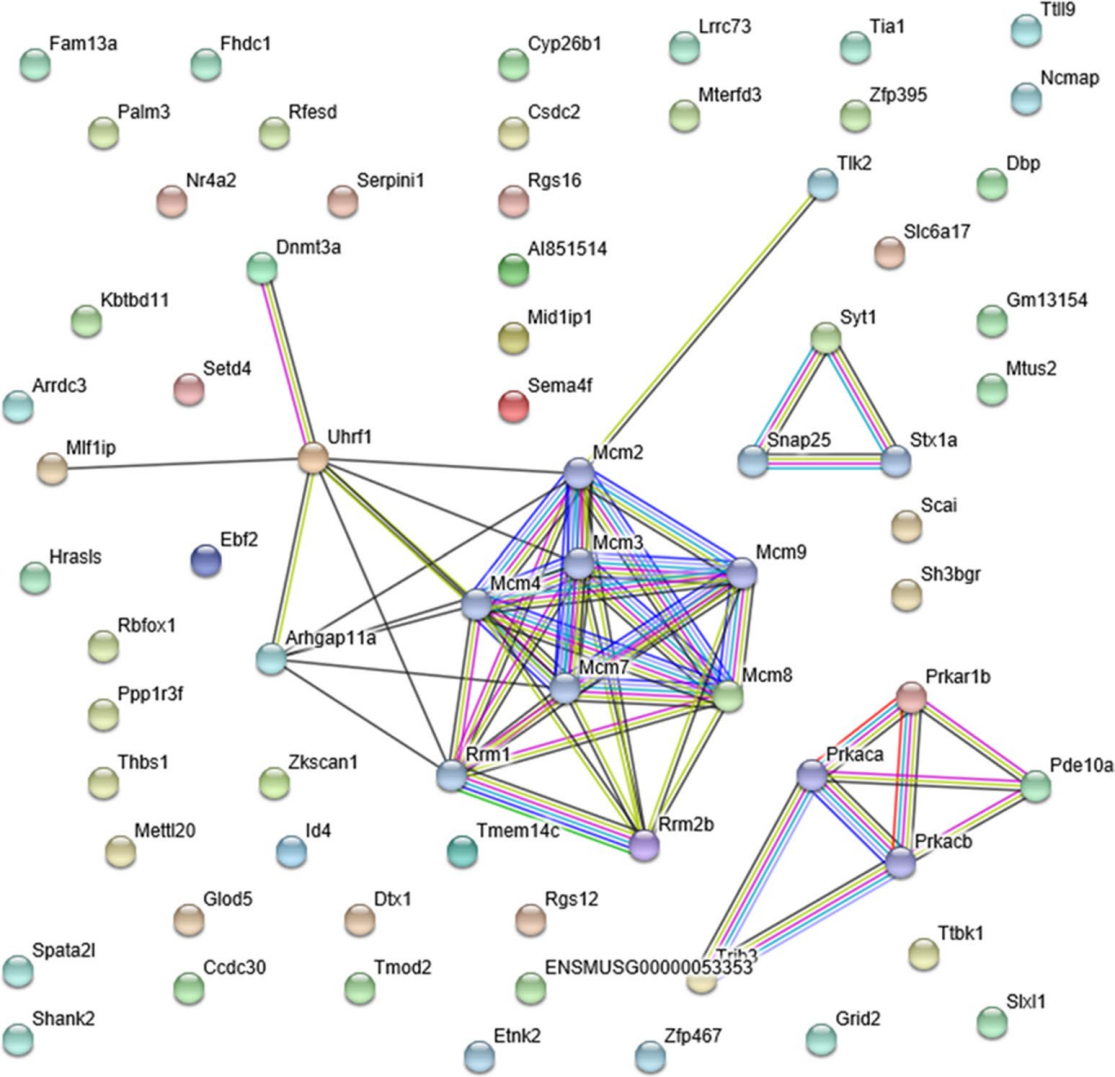
Protein network analysis by STRING software. The information about the restoration of gene expression by SRme administration in hyperlipidemic mice was uploaded into STRING software (version 9.1) for the analysis of the interactions of related proteins and protein–protein interactions. Network nodes represent proteins and edges represent protein–protein associations. Light blue colored edges mean known interactions imported from curated databases; purple, experimentally determined. Green colored edges mean predicted interactions between genes of neighborhood; red, gene fusions; dark blue, gene co-occurrence

By using the STRING database, we assessed functional genomics and explored the predicted interaction networks, which can suggest new directions for future experimental research. In this study, the assessment of 71 genes restored by SRme administration illustrated the changes in pathway activities in liver tissue (Table 3). Pathway analysis suggested that pathways such as DNA replication initiation and DNA helicase activity, and the minichromosome maintenance protein (Mcm) complex had a critical role in the amelioration of hyperlipidemia in mice. Furthermore, the main target proteins with key roles in the aforementioned pathways were identified as Mcm proteins (Fig. 7).

**Table 3:** Functional enrichments in protein network

Pathway ID	Pathway description	Count in gene set	False discovery rate
Biological process (GO)			
GO:0006270	DNA replication initiation	4	0.00262
GO:0006268	DNA unwinding involved in DNA replication	3	0.0227
Molecular function (GO)			
GO:0003678	DNA helicase activity	4	0.00299
GO:0004386	Helicase activity	6	0.00299
GO:0032559	Adenyl ribonucleotide binding	16	0.00299
GO:0036094	Small molecule binding	21	0.00299
GO:0000166	Nucleotide binding	19	0.00685
GO:0004691	cAMP-dependent protein kinase activity	2	0.00951
GO:0004748	Ribonucleoside-diphosphate reductase activity, thioredoxin disulfide as acceptor	2	0.00951
GO:0097367	Carbohydrate derivative binding	18	0.00951
GO:0005524	ATP binding	14	0.0139
GO:0043168	Anion binding	19	0.0149
GO:0051018	Protein kinase A binding	3	0.023
Cellular component (GO)			
GO:0042555	MCM complex	6	2.29e−10
GO:0005952	cAMP-dependent protein kinase complex	3	0.00123
GO:0097362	MCM8-MCM9 complex	2	0.00495
GO:0070033	Synaptobrevin 2-SNAP-25-syntaxin-1a-complexin II complex	2	0.0111
GO:0070032	Synaptobrevin 2-SNAP-25-syntaxin-1a-complexin I complex	2	0.0178

GO terms and pathways associated with differentially expressed genes of liver tissues in hyperlipidemic mice. False discovery rate corrections were calculated using the Benjamini–Hochberg procedure

## Discussion

Salviae miltiorrhizae Radix, the dried root of *S. miltiorrhiza*, is one of the most well-known medicinal resources in Asian traditional medicine [10–14, 17]. Many studies have been conducted on SR, which have provided information on its traditional uses [22], chemical constituents [23, 24], and pharmacological effects [15, 25, 26]; however, the identification of molecular targets and specific effects is still required.

Recently, many researchers conducted studies on tanshinone IIA, one of well-known pharmacologically active components of SR, and demonstrated its involving in intake and efflux of cholesterol, therapeutic potential on cardiovascular diseases such as atherosclerosis [27–30]. Furthermore, tanshinone IIA was reported to have effects stabilizing vulnerable plaques in apolipoprotein-E-deficient (apoE−/−) mice [31].

In Asian traditional medicine, whole plants or mixtures of several plants are used rather than isolated compounds. The aim of this study is to investigate anti-hyperlipidemic effects of SRme in mice model, and deduce molecular target of SRme by evaluation of gene expression in hepatic tissues.

In this study, it was shown that SRme administration significantly decreased triglyceride content without alteration of body weight in mice (Figs. 2, 3c). In our preliminary study, food intake was observed to exclude the possibility that the incorporation of SRme into rodent chow affected food intake and subsequently influenced the changes in body weight and total lipid content in blood. However, it was found that SRme mixed chow did not affect food intake; therefore, it may contribute to the restoration of body weight among the experimental groups of mice. Although the content of MDA was not significantly altered by the administration of SRme, the levels tended to decrease (Fig. 4).

Through the evaluation of hundreds of differentially expressed genes in hyperlipidemic mice, we identified key molecular pathways that play important roles in DNA replication (Table 3); using another database, similar results were observed (Additional file 5: Table S1).

By using a protein network database such as STRING, we identified target proteins, including Mcm proteins, which play a key role in DNA replication (Fig. 7). These results support the data from molecular pathway identification (Table 3). Mcm proteins are known as essential replication initiation factors, and orchestration of the functional interactions between Mcm proteins results in initiation of DNA synthesis in cell cycle [32]. Names of genes which are functionally important in Fig. 7 are provided in Additional file 5: Table S2. One of critical limitations of our study is lack of investigating meaningful relationship between biochemical and genomic data. Furthermore, the currently identified targets of SRme in hyperlipidemic mice are relatively broad, and still not clearly explored. However, based on the present study, we hope the limitation of our study will be overcome through our future researches.

Collectively, the results showed that SRme suppressed hyperlipidemia and the accumulation of triglycerides. In addition, we proposed that the effect of SRme on hyperlipidemia occurred through the restoration of expression of genes and proteins related to DNA replication.

## Conclusions

In order to ascertain the influence of SRme in hyperlipidemic HFD mice, the changes in serum lipids and gene expression were observed. There was no significant effect on the body weight gain in hyperlipidemic HFD mice. The blood serum level of triglycerides induced by hyperlipidemia was restored to that of non-hyperlipidemic mice. Mcm proteins were identified as molecular targets that play a key role in the amelioration of hyperlipidemia.

## Additional files

**Additional file 1.** The minimum standards of reporting checklist.

**Additional file 2.** HPLC images of SR and its standard compound, salvianol acid B (Fig. S1).

**Additional file 3.** Hierarchical clusters of genes which was analyzed using a multiple experiment viewer (Fig. S2).

**Additional file 4.** Fold changes of microarray data set. A microarray containing approximately 45,000 oligo-spots conducted.

**Additional file 5.** Functional enrichments in protein network obtained from KEGG (Table S1), and symbols of functionally important genes from Fig 7, and its descriptions (Table S2).

## Abbreviations

SR: Salviae miltiorrhizae Radix
SRme: methanol extract of Salviae miltiorrhizae Radix
HDL-cholesterol: high density lipoprotein
MDA: malondialdehyde
