# Supplementary figures and images for: Effect of methanol extract of Salviae miltiorrhizae Radix in high-fat diet-induced hyperlipidemic mice
Source: Chin Med. 2017 Oct 13;12:29. doi: 10.1186/s13020-017-0150-0 (PMC5640945; doi:10.1186/s13020-017-0150-0)

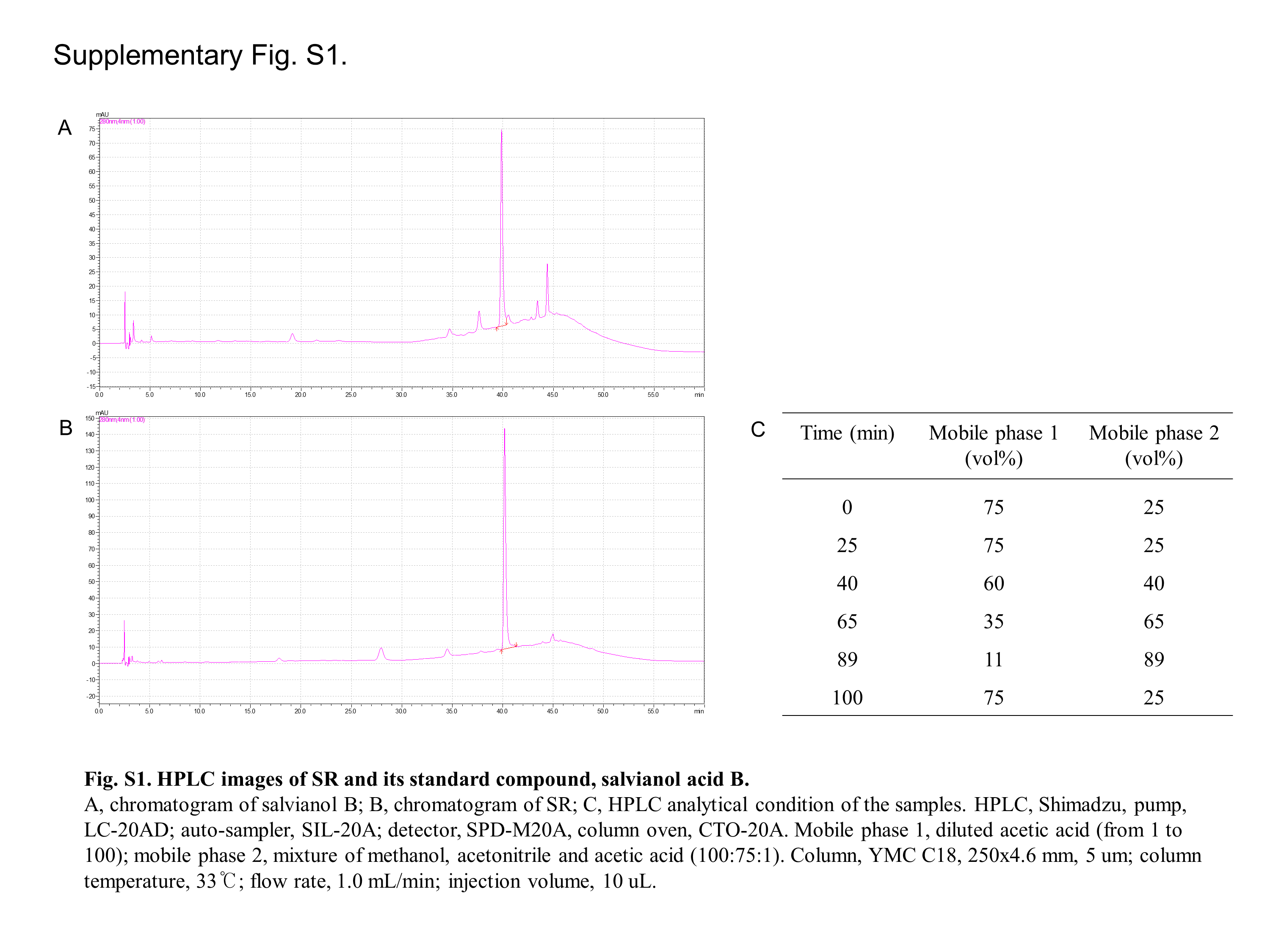

Supplement: Supplementary file 2 — Additional file 2. HPLC images of SR and its standard compound, salvianol acid B (Fig. S1). [file 13020_2017_150_MOESM2_ESM.tif]

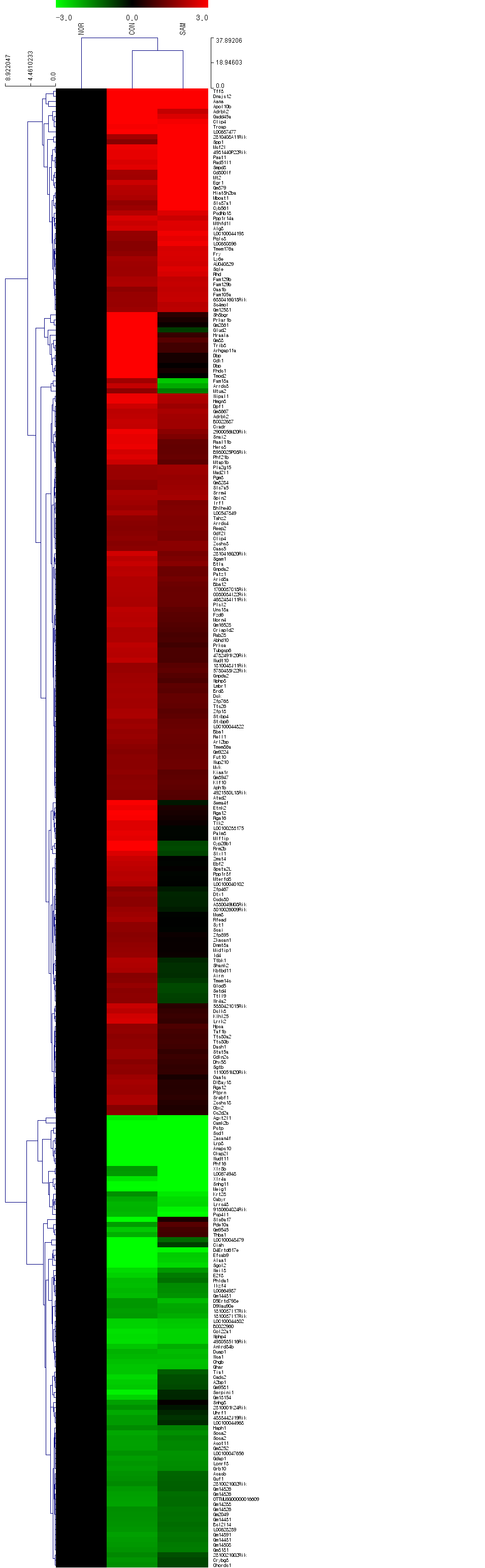

Supplement: Supplementary file 4 — Additional file 4. Fold changes of microarray data set. A microarray containing approximately 45,000 oligo-spots conducted. [file 13020_2017_150_MOESM4_ESM.tif]
