# Supplementary material for: Effect of methanol extract of Salviae miltiorrhizae Radix in high-fat diet-induced hyperlipidemic mice
Source: Chin Med. 2017 Oct 13;12:29. doi: 10.1186/s13020-017-0150-0 (PMC5640945; doi:10.1186/s13020-017-0150-0)
Supplement: Supplementary file 5 — Additional file 5. Functional enrichments in protein network obtained from KEGG (Table S1), and symbols of functionally important genes from Fig 7, and its descriptions (Table S2). [file 13020_2017_150_MOESM5_ESM.docx]

**Supplementary Table S1. Functional enrichments in protein network obtained from KEGG**

| KEGG Pathways | | | |
| --- | --- | --- | --- |
| Pathway ID | Pathway description | Count in gene set | False discovery rate |
| 03030 | DNA replication | 4 | 0.00115 |
| 04911 | Insulin secretion | 4 | 0.019 |
| 04110 | Cell cycle | 4 | 0.0443 |
| 04721 | Synaptic vesicle cycle | 3 | 0.0443 |
| 04910 | Insulin signaling pathway | 4 | 0.0443 |
| 05031 | Amphetamine addiction | 3 | 0.0443 |

**Pathways associated with differentially expressed genes of liver tissues in hyperlipidemic mice.** False discovery rate corrections were calculated using the Benjamini-Hochberg procedure.

**Supplementary Table S2. Symbols of functionally important genes from Figure 7, and its descriptions.**

| **Symbols** | **Descriptions** |
| --- | --- |
| \| Dnmt3a \| \| --- \| \| Mlf1ip \| \| Arhgap11a \| \| Mcm8 \| \| Pde10a \| \| Prkar1b \| \| Rrm2b \| \| Syt1 \| \| Tlk2 \| \| Trib3 \| \| Uhrf1 \| | \| DNA (cytosine-5)-methyltransferase 3 alpha \| \| --- \| \| myeloid leukemia factor-1 interacting protein \| \| Rho GTPase-activating protein 11A \| \| Minichromosome Maintenance 8 \| \| Phosphodiesterase 10A \| \| cAMP-dependent protein kinase type I-beta regulatory subunit \| \| Ribonucleoside-diphosphate reductase subunit M2 B \| \| Synaptotagmin 1 \| \| Tousled Like Kinase 2 \| \| Tribbles homolog 3 \| \| Ubiquitin-like, containing PHD and RING finger domains, 1 \| |
